# Pediatric Metastatic Crohn’s Disease of the Liver

**DOI:** 10.1097/PG9.0000000000000107

**Published:** 2021-07-22

**Authors:** Katie R. Conover, Conrad B. Cox, Huiying Wang, Riha Bhatt, Einar T. Hafberg

**Affiliations:** From the * Department of Pediatrics, Monroe Carell Jr. Children’s Hospital at Vanderbilt; † Division of Pediatric Gastroenterology, Hepatology, and Nutrition, Monroe Carell Jr. Children’s Hospital at Vanderbilt; ‡ Division of Pediatric Pathology, Department of Pathology, Microbiology, and Immunology, Monroe Carell Jr. Children’s Hospital at Vanderbilt.

**Keywords:** Crohn’s disease, granuloma, liver disease, hepatitis, metastatic Crohn’s disease, differential

## Abstract

Metastatic Crohn’s disease (MCD) is the manifestation of Crohn’s disease outside of the gastrointestinal tract and most frequently involves mucocutaneous and pulmonary tissues. This is an uncommon phenomenon but is well characterized in the pediatric literature. In contrast, MCD affecting the liver has not previously been described in pediatrics. The pediatric gastroenterologist must be aware of the myriad of Crohn’s disease–associated hepatopathies. We herein present the first reported case of pediatric MCD involving the liver and describe our targeted diagnostic evaluation and the patient’s response to infliximab-dyyb.

## INTRODUCTION

Pediatric patients with Crohn’s disease (CD) are at increased risk for developing significant hepatopathies including primary sclerosing cholangitis, autoimmune hepatitis (AIH), and infectious hepatitis. In addition, several medications used in the treatment of CD can cause drug-induced liver injury (DILI) (1). Hepatopathies are often highly morbid asymptomatic conditions, and biochemical liver enzyme and function monitoring is standard of care in the management of CD. When liver pathology is suspected, a biopsy is helpful to identify the cause.

A noncaseating granuloma in the gastrointestinal tract can be pathognomonic for CD in the appropriate clinical setting. Metastatic Crohn’s disease (MCD) is characterized by the development of noncaseating granulomata within extraintestinal tissues, most commonly mucocutaneous or pulmonary. Some reports in adult patients describe hepatic granulomas as a manifestation of MCD (2–4). We report a case of hepatic granulomas resultant of MCD in a pediatric patient with CD. The patient’s legal guardians provided informed consent for publication of the case details.

## CASE PRESENTATION

**FIGURE 1.**
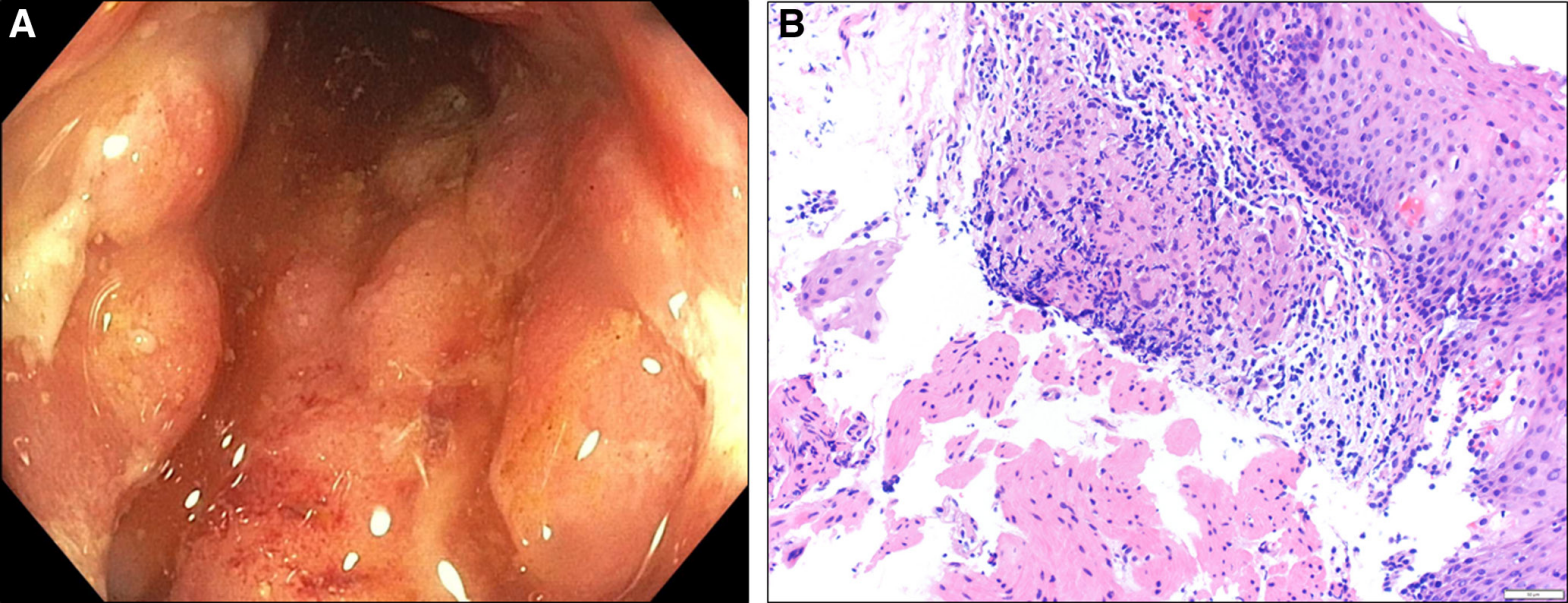
Initial endoscopic and histologic findings highly suggestive of Crohn’s disease. A) Friable pseudo polypoid lesions throughout colon. B) Noncaseating granuloma of the lower esophagus (H&E ×20 magnification). H&E, hematoxylin and eosin.

An 8-year-old male with severe healthcare-associated anxiety presented to the emergency department with protein-calorie malnutrition (body mass index = 4.11), tenesmus, and intermittent hematochezia. He had no fever, cough, sick exposures, or significant travel history. Examination revealed inflamed perianal skin tags (Fig. 1). Initial laboratory evaluation demonstrated severe iron deficiency anemia and hypoalbuminemia, but normal transaminase levels. He underwent endoscopy and cross-sectional imaging which suggested CD A1L4aB1p (Pediatric Paris classification), confirmed by histology showing noncaseating granulomas of the colonic and esophageal epithelium (Fig. 1). Genetic analysis was also preformed due to uncertainty at which age symptoms first arose. Monogenic inflammatory bowel disease and primary immune deficiency panels revealed no pathogenic sequences (Table 1).

**TABLE 1.** Patient laboratory results

Laboratory test	Result
Ceruloplasmin	45 mg/dL (20–60 mg/dL)
Tissue transglutaminase IgG antibody	Negative
IgA quantitative	727 mg/dL (50–220 mg/dL)
C-Reactive Protein	Peak: 85 mg/L (0–1 mg/L) Most recent: 4.7 mg/L (0–1 mg/L)
Erythrocyte sedimentation rate	Peak: 115 mm/h (1–33 mm/h) Most recent: 53 mm/h (1–33 mm/h)
Fecal lactoferrin	Peak: 949.2 μg/mL (<30 μg/mL) Most recent: <30 μg/mL (<30 μg/mL)
Antinuclear antibody	Negative
Antimitochondrial antibody	Negative
Liver-kidney-microsome IgG antibody	<1:20
F-actin IgG antibody	Equivocal
IgG quantitative	1695 mg/dL (540–1360 mg/dL)
Hepatitis B surface antigen	Negative
Hepatitis B core antibody	Negative
Hepatitis B surface antibody	Negative
Angiotensin-converting enzyme	37 U/L (24–121 U/L)
2-view chest radiographs	Normal Hilar Silhouette
QuantiFERON gold	Negative
NADPH activity	Normal
Invitae IBD and PID panels	Negative

IBD = inflammatory bowel disease; NADPH = nicotinamide adenine dinucleotide phosphate; PID = primary immunodeficiency.

He began intravenous methylprednisolone, olanzapine, and nasogastric tube feeds. His treatment course was complicated by refeeding syndrome. Additionally, within 1 week of admission, he developed a noncholestatic hepatitis (alanine aminotransferase and aspartate aminotransferase 4 times upper limit of normal) with preserved synthetic function. Following this, he received infliximab-dyyb (INX) (5 mg/kg) due to inadequate response to corticosteroids, and within 1 week, his diarrhea and refeeding syndrome resolved. He was discharged home on a prednisone taper and outpatient INX infusions.

**FIGURE 2.**
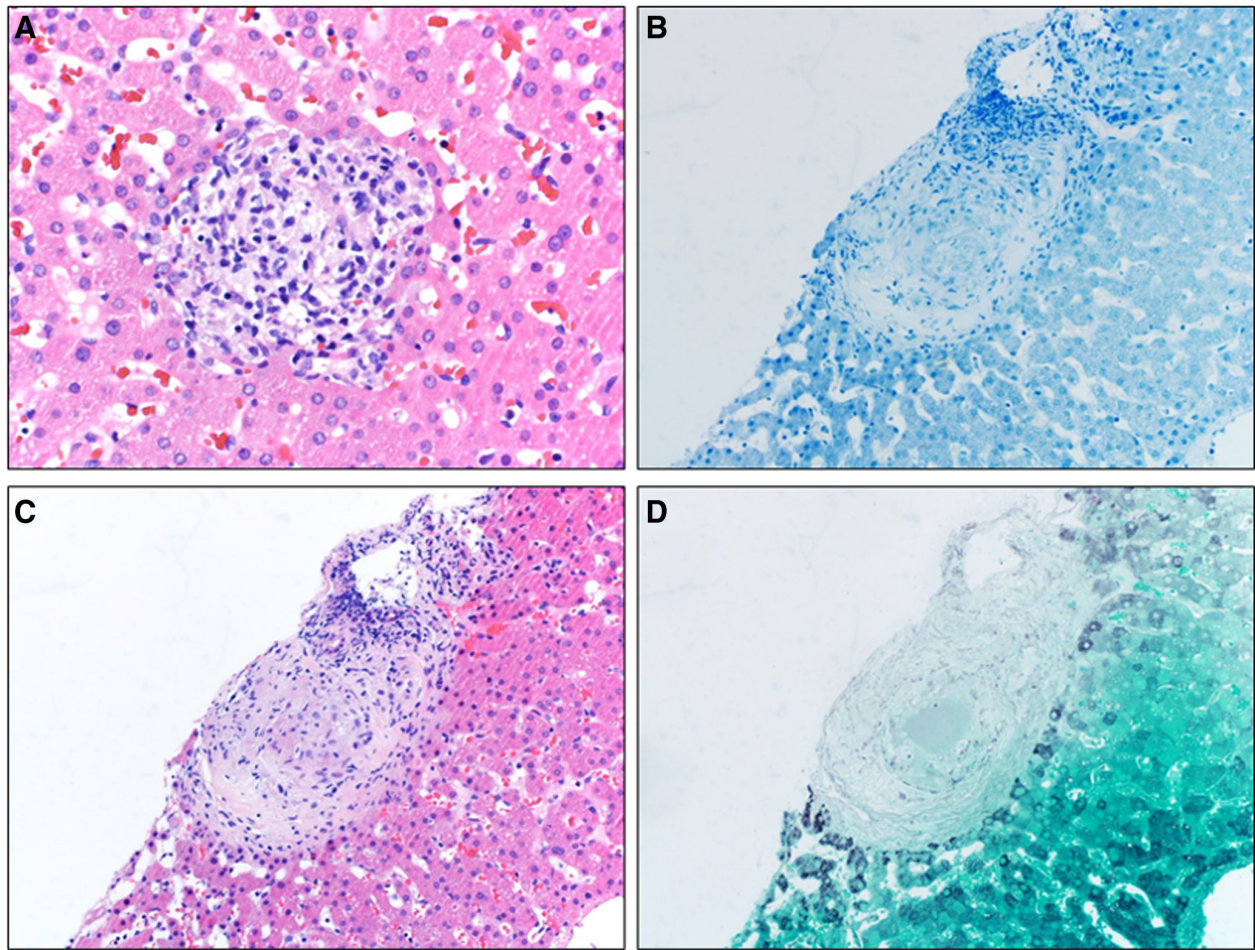
Initial liver biopsy performed due to concern for autoimmune hepatitis was negative for lymphoplasmacytic infiltrate. A) Lobular noncaseating granuloma (H&E ×400 magnification). B) Periportal noncaseating granuloma (H&E ×20 magnification). C) Grocott’s methenamine silver stain negative for fungal organisms. D) Ziehl-Neelsen staining negative for mycobacterial organisms. H&E, hematoxylin and eosin.

Transaminase levels normalized, and his general state of health improved over the following 5 months. However, despite strict adherence to INX infusions and eventual dose escalation (10 mg/kg every 6 weeks), trough concentrations decreased with time, and he developed recurrence of symptoms. He was readmitted and underwent INX reinduction and dose escalation to 15 mg/kg every 4 weeks. Before reinduction, transaminases were again elevated, 2 times upper limit of normal. Additional labs revealed a moderately elevated total IgG and an equivocal F-actin. Liver biopsy for possible AIH revealed 2 foci of noncaseating granulomas (1 periportal and 1 lobular), and no evidence of AIH or primary sclerosing cholangitis (Fig. 2). Further evaluation reasonably excluded conditions such as chronic granulomatous disease (CGD), sarcoidosis, and tuberculosis (Table 1).

He remained well on escalated infliximab dosing. Transaminase levels slowly normalized over time. However, despite escalated INX dosing, trough levels remained below 10 μg/mL. A 1-year-follow-up liver biopsy demonstrated resolution of granulomata and was otherwise normal.

## DISCUSSION

Clinicians who care for pediatric patients with inflammatory bowel disease on tumor necrosis factor (TNF)-α inhibitors frequently encounter mildly and transiently elevated transaminase levels (5). In contrast, severe or persistent elevated transaminase levels are suggestive of a de novo hepatopathy, and these cases warrant liver biopsy in addition to laboratory evaluation. In our patient, we unexpectedly discovered 2 foci of noncaseating granulomas on liver biopsy, which directed the differential diagnosis. Culver et al (6) report that the most common causes of hepatic granulomas in the developed world include sarcoidosis, drugs, infections, and neoplasms. For this patient, we also considered MCD, primary biliary cirrhosis (PBC), and CGD.

Pathologists can help identify the specific histologic variant of granuloma, which further guides the differential diagnosis. This patient had noncaseating granulomas, more commonly seen in sarcoidosis, fungal, and other infections, MCD, CGD, and PBC. Interestingly, some reports describe the paradoxical development of sarcoidosis in patients using TNF-α inhibitors (7).

The biochemical analysis and patient profile were helpful to differentiate these pathologies. Tests listed in Table 1 helped exclude sarcoidosis (normal angiotensin-converting enzyme level and normal chest x-ray), CGD (normal nicotinamide adenine dinucleotide phosphates-oxidase activity), and PBC (normal antimitochondrial antibody). PBC is exceptionally rare in pediatrics. TNF-α inhibition placed him at risk for disseminated infections. Thus, negative special stains for acid fast bacilli and fungal organisms were reassuring (Fig. 2). At this point, MCD became the focus of our differential diagnosis.

We remained cognizant of the possibility that this patient had developed overlapping AIH or DILI. An equivocal F-actin and elevated total IgG suggested AIH, but additional autoimmune serologies and histologic evidence satisfactorily excluded this diagnosis. Further, liver histology did not demonstrate known patterns of injury associated with olanzapine or infliximab, so DILI was excluded (1).The patient remained on these medications and underwent repeat liver biopsy at 1 year, which documented resolution of granulomas. Repeat colonoscopy was significantly improved with only scant aphthae. These findings supported the diagnosis of MCD in our patient.

This represents one of the first reported pediatric cases of hepatic granulomas resultant of MCD. Our patient presented with asymptomatic liver enzyme elevation, consistent with a quarter of all patients with hepatic granulomas (4). The limited literature favors extraintestinal disease acting independently from gastrointestinal disease; however, infliximab has been used successfully to treat both (8,9). Over the course of 1 year, monotherapy with high dose INX gradually led to biochemical and histologic resolution of this patient’s hepatic granulomas. Further study is needed to learn more about the prevalence of this entity in pediatric patients with CD, the consequences of this condition if unidentified, and whether INX is the best therapy for MCD with hepatic involvement.

## ACKNOWLEDGMENTS

K.R.C. contributed to the literature review, contributions to conception of the work, primary author, critical revision, and final approval of version being submitted, guarantee that all individuals who meet *JPGN*’s authorship criteria are included as authors of this paper. C.B.C. contributed to the case review, contributions to conception of the work, secondary author, critical revision, and final approval of version being submitted. H.W. contributed to the pathology review, acquisition of pathology slides for inclusion, critical revision and final approval of version being submitted. R.B. contributed to the conception of the work, critical revision, and final approval of version being submitted. E.H. contributed to the conception of the work, critical revision, and final approval of version being submitted.
